# Supplementary material for: Comprehensive blueprint of Salmonella genomic plasticity identifies hotspots for pathogenicity genes
Source: PLoS Biol. 2024 Aug 7;22(8):e3002746. doi: 10.1371/journal.pbio.3002746 (PMC11305592; doi:10.1371/journal.pbio.3002746)
Supplement: S2 Fig — (A) Prevalence of virulence factors, antibiotic resistance (ABR) genes, stress resistance genes, and defence systems on different prophage genera. (B) Prevalence of virulence factors, ABR genes, stress resistance genes, and defence systems on different plasmid incompatibility groups. For (A) and (B), prevalence values shown represent the relative percentages of pathogenicity factors found in prophages/plasmids, indicating the proportion of these factors present within each specific prophage/plasmid class. The data underlying this figure can be found in S2, S4, and S9 Tables. (PDF) [file pbio.3002746.s002.pdf]

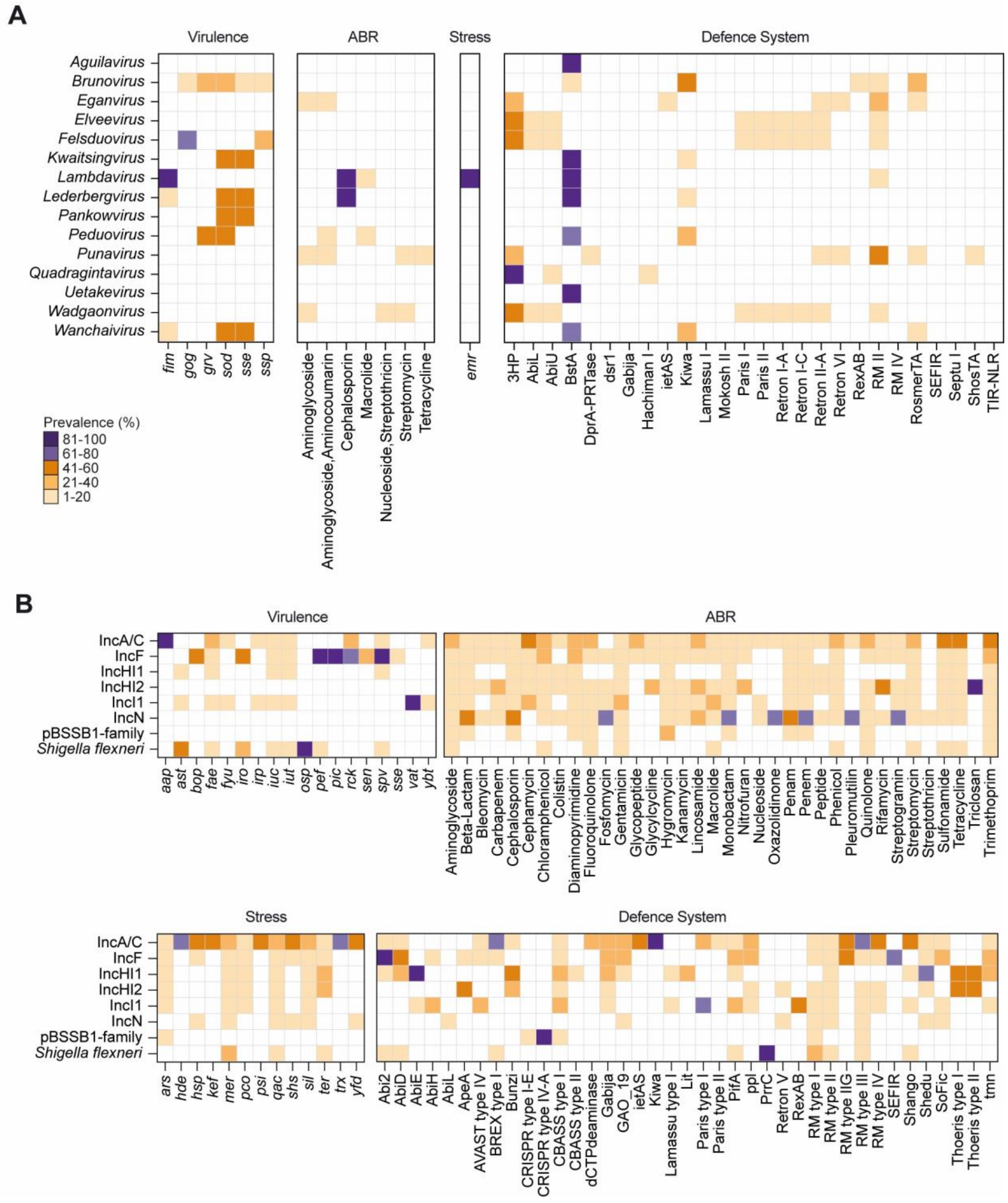

S2 Fig. Distribution of pathogenicity determinants on prophage and plasmid classes. (A) Prevalence of virulence factors, antibiotic resistance (ABR) genes, stress resistance genes, and defence systems on different prophage genera. **(B)** Prevalence of virulence factors, ABR genes, stress resistance genes, and defence systems on different plasmid incompatibility groups. For (A) and (B), prevalence values shown represent the relative percentages of pathogenicity factors found in prophages/plasmids, indicating the proportion of these factors present within each specific prophage/plasmid class. The data underlying this Figure can be found in S2, S4, and S9 Tables.
