# Supplementary material for: Comprehensive blueprint of Salmonella genomic plasticity identifies hotspots for pathogenicity genes
Source: PLoS Biol. 2024 Aug 7;22(8):e3002746. doi: 10.1371/journal.pbio.3002746 (PMC11305592; doi:10.1371/journal.pbio.3002746)
Supplement: S1 Fig — The prevalence of the specific gene cluster in chromosome (black), prophage (white), or plasmid (grey) is shown as a bar graph. The data underlying this figure can be found in S7 Table. (PDF) [file pbio.3002746.s001.pdf]

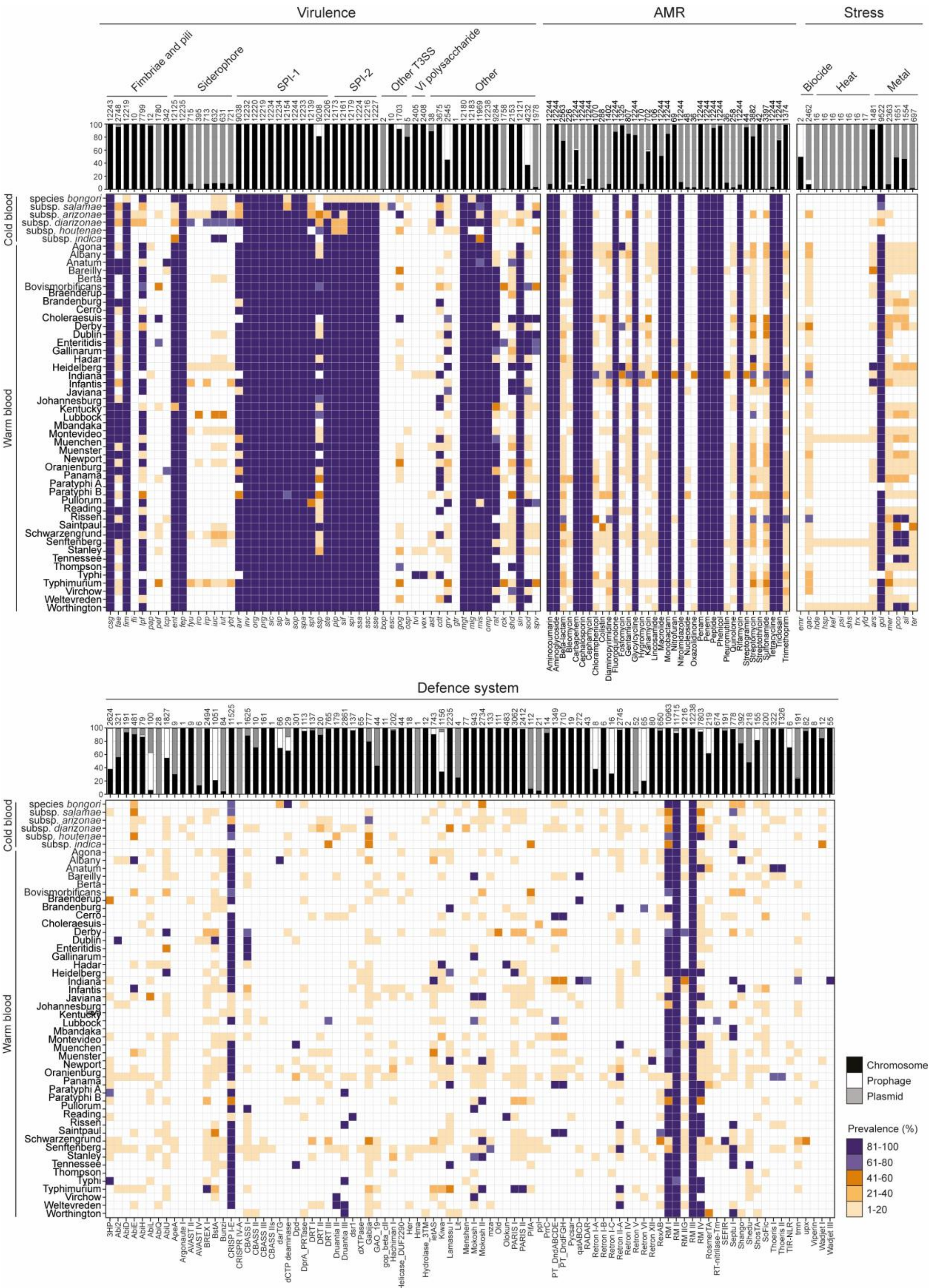

S1 Fig. Distribution of virulence factors, antibiotic resistance (ABR) genes, stress resistance genes, and defence system across the *Salmonella* genus. The prevalence of the specific gene cluster in chromosome (black), prophage (white), or plasmid (grey) is shown as a bar graph. The data underlying this Figure can be found in S7 Table.
