# Supplementary material for: Comprehensive blueprint of Salmonella genomic plasticity identifies hotspots for pathogenicity genes
Source: PLoS Biol. 2024 Aug 7;22(8):e3002746. doi: 10.1371/journal.pbio.3002746 (PMC11305592; doi:10.1371/journal.pbio.3002746)
Supplement: Attachment — Submitted filename: Response-to-reviewers.docx [file pbio.3002746.s019.docx]

**Academic Editor (Overall)**

As you will see, the reviewers are generally positive about your manuscript, but they raise overlapping concerns that mutational-driven resistances are not addressed in the study. After discussions with the Academic Editor, we agree that the study should be strengthened by analysing resistance by genomic mutations and this could be achieved by using antibiotic resistance databases (such as CARD) to detect such mutations in the Salmonella genome.

We understand the importance of analysing resistance through genomic mutations. In response to the suggestion by the reviewers, we have now conducted an analysis using the Comprehensive Antibiotic Resistance Database (CARD) on 12.2K strains. The revised version of the manuscript incorporates data obtained from both Abricate and CARD.

In addition, Reviewer #1 notes that the Platon prediction tool used is now outdated since numerous tools that display higher accuracy have now been developed. Whilst re-running the whole analysis may be a significant undertaking, we ask that the revised version at least includes a confirmation for a few findings with a more up-to-date tool and discusses the limitations of using the current prediction tool.

Reviewer #1 suggests using the tool Plasmer over Platon. We have now re-analysed the 12.2K strains using Plasmer, and this approach has indeed identified an increased number of plasmid contigs. The revised manuscript now includes this updated plasmid analysis conducted with Plasmer.

**Reviewer Comments**

**Reviewer #1:** The manuscript provides an overview of mobile elements and significant functional genes in Salmonella, exploring genetic diversities of plasmids, prophages, virulence genes, ARGs, and stress response genes. While offering substantial data, it lacks detailed analysis.

Several pieces of information presented in the manuscript are already well-established. For instance, the distribution of most virulence genes in the chromosome and antibiotic resistance genes in plasmids is widely known. Notably, a majority of Salmonella's virulence genes have been previously identified within at least 20 pathogenic islands (SPIs). Although the manuscript discusses SPI-1 and SPI-2, it overlooks others. These SPIs are pivotal as they demonstrate the clustering of virulence genes in the chromosome, akin to the RGPs outlined in the manuscript. It is unclear why these important previous researches had been ignored completely.

We appreciate the reviewer’s interest in a comprehensive analysis of all 24 *Salmonella* pathogenicity islands. However, as of now, no tool is available that can efficiently identify all SPIs in the *Salmonella* genome. Therefore, conducting such an extensive analysis in 12.2k strains is currently not feasible. Instead, we have focused our study on the two well-established pathogenicity islands, SPI-1 and SPI-2.

Additionally, the authors utilized Platon for plasmid prediction. However, this area is dynamic, with numerous new prediction tools emerging in the past 3-4 years, some of which appear to outperform Platon, as evidenced by benchmark comparisons (for instance, see <https://eur03.safelinks.protection.outlook.com/?url=https%3A%2F%2Fjournals.asm.org%2Fdoi%2F10.1128%2Fspectrum.04645-22&data=05%7C02%7Cf.nobrega%40soton.ac.uk%7C539013119a844ba2699608dc47451e39%7C4a5378f929f44d3ebe89669d03ada9d8%7C0%7C0%7C638463609387084850%7CUnknown%7CTWFpbGZsb3d8eyJWIjoiMC4wLjAwMDAiLCJQIjoiV2luMzIiLCJBTiI6Ik1haWwiLCJXVCI6Mn0%3D%7C0%7C%7C%7C&sdata=XZ9fNDIn14kclrSIvj965rf1zJLwQZsZqMZWaoBqNUg%3D&reserved=0>).

We thank the reviewer for this suggestion and for pointing us towards the benchmark comparison. This comparison suggests using the tool Plasmer over Platon. We have now re-analysed the 12.2K strains using Plasmer, and this approach has indeed identified an increased number of plasmid contigs. The revised manuscript now includes this updated plasmid analysis conducted with Plasmer.

Concerning antibiotic resistances, the authors neglected to address mutation-driven resistance, particularly quinolone resistance resulting from mutations in QRDR. This form of resistance is particularly worrisome in Salmonella and is prevalent across various serovars, including Paratyphi A, despite the manuscript's description of this serovar as having a "minimal presence of ABR genes."

We agree with the reviewer about the importance of analysing resistance through genomic mutations. We have now conducted an analysis using the Comprehensive Antibiotic Resistance Database (CARD) on 12.2K strains. The revised version of the manuscript incorporates data obtained from both Abricate and CARD.

**Reviewer #2:**

The paper is well written and describes a broad study of Salmonella genomes in the context of the prevalence of gene clusters relating to virulence, antibiotic resistance, stress and anti-phage defence systems. The authors observe cases where specific regions of the genome play host to specific gene clusters and give examples of functional interplay between the chromosome and the gene cluster. The data presented suggest that most gene clusters are not found in spots, and this angle could be explored in greater depth in the manuscript, to suggest why some gene clusters are spot-specific and others are more generalist.

We thank the reviewer for the positive feedback and insightful suggestions. We have provided detailed responses to the comments below, including addressing the comment related to most gene clusters not being found in spots (point #11).

The presence/absence of determinants is the main thrust of this work, which is of key importance and is well documented. However, it is also the case that mutations will impact upon virulence and antimicrobial resistance, and this is not discussed. The presence of a determinant does not necessarily equate to a functional protein or even an expressed gene, a nuance that is extremely relevant to "shaping the pathogenicity, adaptation and distribution of Salmonella". This becomes a further issue when discussing multidrug resistance (e.g. line 195-197). As written, this suggests that only serovars Indiana and Rissen harbour multidrug resistance. As mutational resistance is not considered, this view of MDR is only a partial one, which should be acknowledged.

We agree that it is important to analyse resistance through genomic mutations. We have now conducted an analysis using the Comprehensive Antibiotic Resistance Database (CARD) on 12.2K strains. The revised version of the manuscript incorporates data obtained from both Abricate and CARD.

Additional points are covered below:

1.      Line 80-82. Pearce et al (citation 85) have proposed an updated range of Salmonella species and subspecies, suggest to rephrase to reflect this.

Following the recommendation mentioned by Pearce et al., we recalculated the Average Nucleotide Identity (ANI) scores by comparing them with the type strains *S. bongori* (NCTC 12419), *S. enterica* subsp. *arizonae* (NCTC 8297), *S. enterica* subsp. *enterica* (NCTC 12416), *S. enterica* subsp. *diarizonae* (NCTC 10060), *S. enterica* subsp. *salamae* (NCTC 5773), *S. enterica* subsp. *indica*, VI (NCTC 12420) and *S. enterica* subsp. *houtenae*, IV (NCTC 12418). Strains with ANI 95% were retained. Methods and figures were updated in the revised manuscript.

2.      Line 85. Whilst Typhimurium is a main causative agent of gastroenteritis, suggesting that all 2170 strains are from human gastroenteritis is a vast oversimplification - there will be representation from invasive disease as well, and animal disease. The methods (line 550-3) state that host specificity was designated based upon literature review. Was strain specific metadata associated strains in PATRIC and NCBI used to help with this designation? Were entire serovars designated with a single host specificity (as suggested in Fig 1A)? If so, again this is an oversimplification for serovars like Typhimurium and Enteritidis.

We appreciate the reviewer’s attention to detail, and we have made the following modifications to address the concerns raised:

- We revised the statement suggesting that all 2170 Typhimurium strains are from human gastroenteritis to “As expected, the most prevalent serovars were Typhi (2,440 strains) and Typhimurium (2,170 strains), the main causative agents of typhoid fever in humans (14) and typhoid fever/gastroenteritis (15) in various animals and humans, respectively.”
- We have indeed designated host specificity for the entire serovar. To avoid oversimplification, we have removed the host range classification from the manuscript and figures.

3.      Line 101-105. There is a distinction to be made here between contig number and identified type. Could a higher contig number mean a larger plasmid, rather than more plasmids?

Yes, a higher contig number can indicate a larger plasmid or the presence of multiple plasmids. The analysis specifically focuses on counting plasmid contigs, which may not always accurately reflect the actual count of plasmids in the strains under consideration. We made modifications to the text to make this aspect clear to the readers:

“Analysis of plasmid prevalence across different *Salmonella* subspecies and serovars revealed a variable abundance of the plasmid contigs (i.e., contigs that represents a part or the entirety of a plasmid) (**Fig. 1c, S2 Table**).”

4.      Line 145. Why is it curious that SPI-1 is prevalent across all Salmonella? This has been reported previously and its presence indicates it is ancestral to the genus.

Indeed, this is a well-known fact, so the word “Curiously” has been removed in the revised manuscript.

5.      Line 149-153. Enteritidis is known to harbour a different virulence plasmid to Gallinarum/Pullorum (<https://doi.org/10.1073/pnas.1416707112>), which the rck evidence supports.

We have added this information to the revised manuscript text as follows:

“Serovars Gallinarum and Pullorum share a more recent common ancestor with the serovar Enteritidis, but they harbour a different virulence plasmid (19). This difference is evidenced by the absence of gene *rck*, which is responsible for evading the host immune response and surviving inside the host (20), in both Gallinarum and Pullorum virulence plasmid (**S1 Fig**).”

6.      Line 205-207. There is an overreaching assertion here that this study shows that the antibiotic resistance observed in S. enterica subsp. enterica is due to human antibiotic usage. Whilst this is a reasonable hypothesis, this work does not provide evidence for this assertion (e.g. that the resistance determinants identified correlate in some way with human antibiotic usage more than e.g. animal husbandry or food production) and it should be rephrased to make it clear this is a hypothesis.

We recognize that the sentence could be misleading, so we have removed it.

7.      Similar to point 6, line 236-238 makes a comment about animal production settings. Is there associated metadata with the strains to suggest that mentioned "specific serovars" are associated with animal production?

As for point 6, we have removed these sentences from the revised manuscript.

8.      Line 301-304. Please clarify what "the genes" refer to. This sentence refers to Figure 3A, the legend of which refers to gene families. Which is it? Figure 3B refers to genes again.

The pangenome analysis representing the gene repertoire showed 77,509 gene families in 12.2K *Salmonella* strains. In the revised manuscript, we adjusted the text so that the terminology used in this context is gene families.

9.      Section starting line 294. The term 'persistent' has specific biological meaning, and has been applied to describe Salmonella strains that persist e.g. in the presence of antibiotic selective pressure. To clarify the use of 'persistent genes' here, it would be good to have a clear explanation of this term in the context of gene variation.

The term "persistent" refers to the gene families present in 90-99% of the strains under consideration. This definition has been added to the revised manuscript as follows:

“The pangenome analysis categorises gene families as core (conserved in all genomes), persistent (found in more than 90% of genomes), shell (present in 50% to 90% of genomes), and cloud (found in less than 50% of genomes). Our findings show that only 4.6% of the gene families are conserved in over 90% of *Salmonella* genomes (3,575 persistent gene families, including 65 core families), while 5.5% of the gene families (4,256) were present at intermediate frequencies (shell), and ~90% (69,678) at low frequency (cloud) (**Fig. 3a**).”

10.     Lines 351-3 and 356-7. In the cases where spot presence does not correlate to expected resistance determinants, is this because the spot is occupied with alternative determinants or remains unoccupied? Is there potential for exclusion of expected determinants if alternative ones are present?

We appreciate the reviewer’s insightful comment. Where spot presence does not correlate with expected resistance determinants, it could indeed be due to the spot being either unoccupied or occupied with alternative determinants. Although we have not specifically analysed this aspect in the current study, it is possible that the presence of certain determinants may exclude others. However, it is also possible for multiple determinants to coexist in the same spot, as demonstrated in our findings. This aspect warrants further exploration in future studies focusing on the dynamics and potential competition among RGP that occupy the same genomic spot. Additionally, determinants might be specific to certain RGP that localise to particular spots, potential creating an exclusionary relationship between different determinants. We agree that this presents an interesting avenue for further research to better understand these interactions and dynamics.

11.      Line 373-378, Figure 4B and line 476-8. The figure indicates that most gene clusters are found outside of spots, and in a broader sense, only a handful of gene clusters are specifically associated with spots. This is really interesting to see, that not all gene clusters behave the same way, as some have a much greater spot specificity than others. This should be presented in greater detail - which gene functions are non-spot specific, and what might that say about their biology?

We thank the reviewer for the insightful suggestions. We agree that exploring the reasons behind the spot-specific and generalist nature of gene clusters is crucial. While our current analysis of spot flanking genes provides some insights, a detailed discussion on this topic remains speculative at this stage. However, we have added a paragraph to the section “Gene clusters integrate into preferential spots in the Salmonella genome” to address why most gene clusters are not (or rarely) found in spots:

“In our study, we also observed that the majority of gene clusters are not associated with specific genomic spots (Fig. 4c). This lack of association can be attributed to several factors. Firstly, many gene clusters, including some responsible for stress resistance (e.g. qac, mer, ter) and antibiotic resistance (e.g. β-lactam, chloramphenicol, streptomycin, among others), are predominantly located on plasmids rather than chromosomal spots (S1 Fig). Plasmids often serve as vectors for horizontal gene transfer, contributing to their widespread distribution across different strains without a fixed genomic position. Additionally, virulence genes such as spv, fyu, irp, and ybt, along with defence systems like PifA, Gao_19, and Bunzi also frequently reside on plasmids, further explaining their absence from chromosomal spots (S1 Fig). Conversely, certain gene clusters, such as sop and ssp from SPI-1 (18) and defence systems RMII and RMIII (S1 Fig) are ubiquitous in *Salmonella* genomes. Their consistent presence across various strains likely precludes their identification in variable spots in our analysis.”

12.     Line 465/141. I couldn't find the accession number for the S. bongori strain that harbours SPI-2 on NCBI - what does this number refer to?

The accession number corresponds to the PATRIC database (<https://www.bv-brc.org/search/?keyword(%221173775.3%22>). We indicate this in the revised text.

13.     Line 535. Typo – potential

This is corrected in the revised manuscript.

14.     Line 561-2. RGP without a corresponding spot were excluded from the analysis - what proportion of the data did this represent? And if it was a substantial amount, how might this affect the conclusions?

Our analysis identified a total of 673,113 RGP, with 71.4% (480,486) clustered in 1,345 spots. RGP not associated with a spot mostly represent contigs which were partially or completely part of an RGP or were plasmid contigs identified by the panRGP tool due to their variable presence among *Salmonella* strains. As these do not fit the criteria for true RGP, their exclusion from the analysis does not impact on our conclusions. We revised the text to incorporate this information:

“Our analysis identified a total of 673,113 RGP, among which 71.4% (480,486) were clustered in 1,345 spots. The RGP not assigned to a specific spot on the chromosome, 95% did not have at least one border (left or right) missing and 20% of them were plasmid contigs, which we excluded from further analysis as they do not meet the criteria for true RGP. The RGP associated with chromosomal spots have an average length of 11,182 bp (range: 3,001-355,687 bp) and an average of 11 genes (range: 4-522) per RGP (**Fig. 3d**).”

15.     Line 571. Please include the versions used for these databases

The dates on which the databases were downloaded are incorporated in the revised manuscript.

Figures
1.      Figure 1C. Does this actually show the average content, or a visual representation of the range of content per subspecies/serovar?

Indeed, Fig. 1c represents the range of plasmid contigs, and Fig. 1F shows the range of prophage content. This has been specified in the graphs and legends of the revised manuscript.

2.      Figure 1E. Does the y-axis represent plasmid contigs or actual plasmid number? Please clarify what 'plasmid number' means.

Fig. 1c is the range of plasmid contigs. We have corrected this in the graph and legend. Of the revised manuscript.

3.      Figure 2A. Why not show the range (like Fig 1)? How uniform are the serovars within themselves?

We have incorporated this suggestion in the revised Fig. 2a. Most variation within serovars is observed for defence systems.

4.      Figure 4A. What do the light grey genes represent?

The light grey genes in Figure 4A represent genes within the RGP that are not the specific pathogenic determinants under study. This is indicated in the revised Fig. 4 and corresponding legend.

**Reviewer #3:** This is an important and innovative analysis of the genomic landscape of the Salmonella genus. The authors have used a new approach to define "regions of genomic plasticity" that have been missed by previous investigations. The study should make an important contribution, not only to the field of Salmonella research, but more broadly to investigations of the genomic landscape of other bacterial genera. However, the manuscript is not yet ready for publication and requires a series of important modifications which are detailed below.

The key findings are based on the concept of genomic plasticity, which will be new to many readers. The authors have cited a paper from 2008 when RGP was first mentioned in the text (Mathee etal 2008, PNAS). However, since 2008, the term RGP has not been widely adopted. Consequently, at Line 49, when the authors first introduce "regions of genomic plasticity" (RGP), they need to explain precisely what the term means. As I understand it, RGP refers to specific contiguous regions of the bacterial genome that are more prone to genetic changes, rearrangements or variations? And the RGP exhibit a higher degree of flexibility and adaptability than the more conserved and stable regions of the genome?

We are thankful for the positive feedback of the reviewer and the constructive criticism provided. We recognise that the terminology "RGP" may not be familiar to all readers, so we have added the following explanation to the text:

“These variable genes are often organised into regions of genomic plasticity (RGP) (5), which are regions of a genome structurally absent in other related genomes and typically associated with frequent rearrangements, such as those mediated by mobile genetic elements (MGEs).”

Much of the text could be written in a clearer and less ambiguous style. For example, Lines 373 - 375 reads:

We appreciate the reviewer’s suggestion and have revised the text to improve clarity at several points.

"On a broader scale, we also observe a general inclination for gene clusters with related functions to cluster within the same spots". Many phrases need to be less vague and written with more clarity.

We revised the sentence as follows:

“On a broader scale, we also observe an inclination for pathogenic determinants with related functions to cluster within the same spots (13 out of 26). This is well demonstrated by spots housing diverse anti-phage defences (e.g., ##11, #39, #43, and #63).”

Another example is at Line 154: "the majority of virulence factors are in chromosomal regions". Clarify, to explain that the majority of genes that encode virulence factors are located on the chromosome, and are not carried by plasmids. Make this statement less vague by stating the percentage of virulence factors that are chromosomally-encoded.

We revised the sentence as follows:

“Our analysis revealed that the majority of genes encoding virulence factors (97%) are located in chromosomal regions, rather than being carried by prophages or plasmids (Fig. 2b, S1 Fig, S6 Table).”

The terms prevalence, average, and frequency are central to the findings, but these terms are used interchangeably. In fact, these terms mean different things. Ensure that each term is used correctly throughout the manuscript text and the figures.

In the revised manuscript we have ensured that the terms "prevalence," "average," and "frequency" are used correctly throughout the manuscript text and figures.

Specific Comments:

Line 22: The Abstract should be rewritten to reflect the key messages of the paper. It should be less generic and vague.

We have revised the abstract as follows:

“Understanding the dynamic evolution of *Salmonella* is vital for effective bacterial infection management. This study explores the role of the flexible genome, organised in regions of genomic plasticity (RGP), in shaping the pathogenicity of *Salmonella* lineages. Through comprehensive genomic analysis, we uncover distinct integration patterns of pathogenicity-related gene clusters into RGP, challenging traditional views of gene distribution. These RGP exhibit distinct preferences for specific genomic spots, and the presence or absence of such spots across *Salmonella* lineages profoundly shapes strain pathogenicity. RGP preferences are guided by conserved flanking genes surrounding integration spots, implicating their involvement in regulatory networks and functional synergies with integrated gene clusters. Additionally, we emphasise the multifaceted contributions of plasmids and prophages to the pathogenicity of diverse *Salmonella* lineages. Overall, this study provides a comprehensive blueprint of the pathogenicity potential of *Salmonella*. This unique insight identifies genomic spots in non-pathogenic lineages that hold the potential for harbouring pathogenicity genes, providing a foundation for predicting future adaptations and developing targeted strategies against emerging human pathogenic strains.”

Line 82; The grouping of serovars into "host-specific, host adapted and broad-host range" should be further described (and discussed). This reviewer does not know a reliable source for this information, it is important to specify the basis of this important categorisation.

We initially designated the host specificity for each serovar based on data from the PATRIC database. However, to avoid oversimplifying this classification, we have removed the host range categorization from the manuscript and figures.

Line 101: specify "plasmid contigs"

The term “plasmid contigs” refers to contigs that represent a complete or partial plasmid. This was specified in the text as follows:

“Analysis of plasmid prevalence across different *Salmonella* subspecies and serovars revealed a variable abundance of the plasmid contigs (i.e., contigs that represents a part or the entirety of a plasmid) (**Fig. 1c, S2 Table**).”

Line 120: one of the most commonly identified phages included Shigella phage Sf6. The significance of a Shigella phage being found in Salmonella isolates should be discussed.

We thank the reviewer for this observation. We would like to clarify that phage identification is based on similarity to known phages, not that the identified phages are exactly the same as those referenced. Therefore, we found that one of the most frequent phages was highly similar to Shigella phage Sf6. This is not surprising given the close phylogenetic relationship between Shigella and Salmonella species. To prevent any confusion, we have revised the text to specify that the phages identified share similarities with the indicated phages.

“The most commonly identified phages show high similarity to *Salmonella* phage SEN34 (S5 Table).”

Overuse of the words "Interestingly" and "Curiously". For example at Line 137, it is expected that there would be fewer virulence genes in S. bongori

"Interestingly" and "Curiously" have been removed from various instances in the revised manuscript.

Line 152: the speculation about rck being involved in the broader host range of Enteritidis needs supporting information. Either add a reference and discuss, or remove.

This sentence was removed.

Line 230: Explain why the Shigella flexneri virulence plasmid is included (and included in figures as well)

We included the *Shigella flexneri* virulence plasmid in our analysis and figures because we used the plasmid typing schemes from PubMLST, which encompass the *Shigella flexneri* plasmid scheme (<https://pubmlst.org/bigsdb?db=pubmlst_plasmid_seqdef&page=query>). This scheme types pINV plasmids into distinct virulence sequence types (vSTs). While pINV plasmids are typically associated with *Shigella flexneri* and enteroinvasive *E. coli* (EIEC) <https://www.biorxiv.org/content/10.1101/2022.10.27.514154v1.full.pdf>), our analysis indicates that homologues of these plasmids are also present in *Salmonella*.

Line 237: which serovars? Are these serovars particularly linked to animals/animal products?

We have removed this line to avoid making unsubstantiated claims.

Line 282: PifA in lower case

Corrected in the revised manuscript.

Line 292: this phrase is vague. The term "differential prevalence" is ambiguous. Which "distinct MGEs" are referred to here?

We have removed this line to avoid making unsubstantiated claims.

Line 301: a good definition of "persistent", "shell", and "cloud" genes is required (there is a brief definition in the legend of Fig 3; this should be moved to text and expanded. What does "almost" mean? The terms "intermediate frequency" and "low frequency" must be defined precisely.

We thank the reviewer for the suggestion. We have expanded the explanation of core, persistent, shell and cloud genes in the text as follows:

“The pangenome analysis categorises gene families as core (conserved in all genomes), persistent (found in more than 90% of genomes), shell (present in 50% to 90% of genomes), and cloud (found in less than 50% of genomes). Our findings show that only 4.6% of the gene families are conserved in over 90% of *Salmonella* genomes (3,575 persistent gene families, including 65 core families), while 5.5% of the gene families (4,256) were present at intermediate frequencies (shell), and ~90% (69,678) at low frequency (cloud) (**Fig. 3a**).”

Line 304: what is the variation in gene length that is shown in Fig 3b? It should be specified in the text.

We have incorporated it in the revised manuscript as follows:

“Analysis of gene length shows that core gene families range from 195 to 3,699 bp, persistent gene families from 93 to 17,610 bp, shell gene families from 93 to 10,137 bp, and cloud gene families from 93 to 24,540 bp (Fig. 3b). The median gene length showed that persistent (873 bp) and core genes (729 bp) are significantly longer than shell genes (558 bp) and cloud genes (420 bp) (Fig. 3b, S9 Table).”

Line 311: define "accessory shell and cloud genes"

We have revised the sentence for clarify as follows:

“Clusters of the less conserved shell and cloud gene families form RGP, which primarily result from horizontal gene transfer events. To study the dynamics of RGP, these regions from different genomes can be grouped into specific insertion spots based on the presence of conserved flanking persistent genes (**Fig. 3c**).”

Line 316: what is the variation in RGP length and number of genes?

The RGP length is of the range 3,001-355,687 bp and gene count is of the range 4-522 genes. We have incorporated it in the revised manuscript as follows:

“The RGP associated with chromosomal spots have an average length of 11,182 bp (range: 3,001-355,687 bp) and an average of 11 genes (range: 4-522) per RGP (**Fig. 3d**).”

Line 334: The difference between spot (#) and hotspot (##) is defined in line 334. However, the two terms seem to be used interchangeably throughout the text and figures. For example, the Y-axis of Fig 4B contains both Spots and Hotspots. Another example is the sentence at Line 415 that begins "Spot ##17 is a hotspot".

We thank the reviewer for pointing out this important detail. We have revised the text to clearly distinguish between spots and hotspots.

Line 334: The definition of a spot lacks detail. The current version is "Spots (#) are characterised by the presence of > 1 and ≤ 100 unique genes". It is important to state that both spots and hotspots are contiguous regions of the chromosome in different Salmonella strains.

The current nomenclature can be improved to improve clarity. Rather than just using "##" in black font to denote hotspots, I suggest that the authors use "##"  in a RED font throughout the text and figures.

We thank the reviewer for this suggestion, which prompted us to realise an inconsistency in our explanation of ‘spot’ and ‘hotspot’ between the text and the figure legend. Spots represent conserved genomic locations where less conserved regions (RGP) integrate. A spot (#) is characterised by the insertion of 1 to 100 unique RGP families in different genomes, while hotspots have more than 100 unique RGP families integrated, suggesting higher rates of gene acquisition. We have rectified this inconsistency in the legend of Fig. 3e, as follows:

“Spots (#) are characterised by the presence of > 1 and ≤ 100 unique RGP families with diverse gene content, and hotspots (**##**) by the presence of > 100 unique RGP families.”

To improve the clarity of the distinction between spots (#) and hotspots (##), we have made “**##**” bold in the text and figures.

Line 373: is it possible to list the other categories?

We thank the reviewer for the feedback as it helped us recognise the lack of clarity in the sentence. Our analysis primarily focused on four categories: virulence, stress resistance, antibiotic resistance, and defence systems. Therefore, the statement refers to the clustering of these pathogenic determinants. All pathogenic determinants identified in the spots are listed in Table S12. We have revised the sentence to improve clarity as follows:

“On a broader scale, we also observe an inclination for pathogenic determinants with related functions to cluster within the same spots (13 out of 26).”

Line 388: which spots and which gene clusters?

The intention of this sentence is to summarize our observation discussed in this section of the text. We present various examples of pathogenic determinants that demonstrate a preference for specific spots analysed here, such as CRISPR-Cas and spot 22, or gold resistance and spot 17. However, we understand the need for clarity and have revised the sentence as follows:

“In summary, our findings reveal that certain pathogenic determinants display a preference for integration into specific spots within the *Salmonella* genome. The presence of these spots indicates the potential presence of the associated pathogenic determinants.”

Line 389: it is speculative to say that "these regions" (RGBs?) play a critical role in bacterial adaptability and fitness as the statement is not supported by experimental evidence.

We have revised the language in the text to reflect that it is a hypothesis and it requires experimental validation.

“These genomic spots may potentially play critical roles in bacterial adaptability and fitness, as evidenced by the exclusive association of the type I-E CRISPR-Cas system with serovars containing hotspot **##**22, warranting future experimental investigation.”

Line 395: is "gene neighbourhood" referring to "gene context"? are "highly conserved genes" persistent or core genes?

Yes, ‘gene neighbourhood’ refers to gene ‘context’, as both terms describe the immediate genomic surroundings of a specific gene. Similarly, ‘highly conserved genes’ refer to persistent and core genes. We have adjusted the sentence to clarify these points:

“We investigated whether the preference of the pathogenicity gene clusters for particular spots was influenced by the gene context, particularly focusing on the highly conserved (persistent and core) genes flanking the spots.”

Line 397: This reviewer would like to see the provision of an interactive resource that would allow individual hotspots to be explored. Have the authors considered this?

We have indeed developed an interactive resource for exploring the genomic content of the hotspots, available on our Github page: <https://simrankushwaha.github.io/Genome-Plasticity-in-Salmonella/>. This resource allows users to investigate the genomic content of each spot across several *Salmonella* strains. However, due to technical constraints, we regret that we are unable to provide individual exploration for each example at this time.

Line 464: The presence of SPI-2 cannot be stated to give S. bongori the ability to infect warm-blooded hosts without experimental evidence.

The hypothesis regarding the role of SPI-2 in providing *S. bongori* with the ability to infect warm-blooded hosts is supported by previous research (<https://doi.org/10.1128/iai.72.5.2879-2888.2004>) where *S. bongori* harbouring a cloned SPI-2 demonstrated the capacity to invade and persist in cells of warm-blooded animals. However, we have revised the sentence to clarify that it is a hypothesis and have included the referenced study.

“Noteworthy examples include the presence of SPI-2 in one strain of *S. bongori*, which may provide it with the ability to infect warm blooded hosts (17).”

Line 469: is #22 referring to hotspot ##22?

Yes. This was corrected in the revised manuscript.

Line 473: vague phrase should be improved.

We have improved the clarity of the text as follows:

“The mobility of gene clusters across genomes has raised questions about their potential integration into sites lacking proper regulation of expression (73-76). Potential issues arise when the cluster relies on regulatory interactions absent in the new host, leading to incorrect gene expression, or when auxiliary interactions and dependencies on the host become relevant (77).”

Line 488: #51 or ##51?

##51. This was corrected in the revised manuscript.

Line 529: This is over-claiming the significance of the study. No mechanistic information can yet be derived from the results.

We have adjusted the language in this paragraph to reflect that further study of gene clusters, RGP, and pathogenic attributes may, in the future, lead to a better understanding of the mechanisms driving the evolution of *Salmonella*.

“In the broader context, these findings offer a novel perspective on deciphering the evolutionary trajectory of *Salmonella*. Further studies focusing on the relationships between gene clusters, RGP, and pathogenic attributes could improve our understanding of the mechanisms driving the emergence of diverse *Salmonella* lineages. Such knowledge might help in predicting future adaptations and developing targeted interventions to combat infections. For example, the identification of genomic spots within strains of non-pathogenic lineages with the capacity to house RGP encoding human pathogenicity genes could help us to foresee evolutionary shifts and formulate preventive strategies against the potential emergence of human pathogenic strains. This approach could involve monitoring these genomic spots and implementing measures to mitigate the risk of pathogenic gene acquisition.”

Line 530: this phrase is speculative and requires support - the authors need to explain how this information could be practically used for designing interventions and to predict evolution.

The sentence was modified as shown in our response to the comment above.

Figures
The beautiful figures are a real highlight of this study. A minor modification will make it easier for the reader to understand the X axis of all figures that include bar charts or similar (Fig 1ACE, Fig 2A, Fig 3E, Fig 4B). Instead of showing text vertically on the page, the text should be angled at 45° to improve readability.

We thank the reviewer for the positive feedback on the figures. While we understand the suggestion to angle the text at 45° on the X-axis of the bar charts for improved readability, we found through testing that this adjustment led to reduced readability. Specifically, the long labels became more overlapped. Therefore, we opted to maintain the current orientation.

Overall, the figure legends need to be substantially improved. Currently they are extremely brief and do not explain how the analysis was actually performed.

We have provided additional detail for each figure legend in the revised manuscript.

Fig 1

Fig 1B: what Is the scale bar? Move the "green" nodes a little to the side to be able to see the distances.
In the legend for figure 1, it needs to be explained that the term average plasmid content and average prophage content refers to the number of plasmids or prophages per genome.
Have the legends for Fig 1E and 1F been swapped by mistake ?

We have updated the figure legend to include the information that the scale bar represents a phylogenetic distance of 0.012 nucleotide substitutions per site. Additionally, we have adjusted the legend to clarify that 1c and 1d denote the range of plasmid contig and prophage count per genome, respectively. Furthermore, we have corrected the labels for 1e and 1f in the revised manuscript. Thank you for bringing these points to our attention.

Fig 3C: This diagram could be improved to aid understanding of RGP organisation. Perhaps an example such as in Fig 4A would work better?

We thank the reviewer for the suggestion. We have adjusted the diagram for better explanation of RGP organisation.

Fig 3D: Need to improve explanation of what this panel shows.

We have improved the explanation in the figure legend, as follows:

**“(D)** Length and gene count in the identified RGP. Each dot indicates an individual RGP length and RGP gene count respectively. Dotted lines indicate the average for each category. ‘n’ indicates the total number of RGP represented.”

Fig 3E: This colour scheme is the same as used for virulence factors, antibiotic resistance, etc in Fig 2B. I suggest a different colour scheme is used for this heatmap.

This is a great suggestion. The colour scheme of Figure 3E has been changed for improved clarity.

The host range categories shown below serovar names in Fig 1A should be added to Fig 3E and a white line across the figure to separate spots and hotspots.

The host range categories (now only cold blooded and warm blooded hosts) were added to Fig. 3e. A dark line was added to separate spots and hotspots.

Fig 4A is not discussed much in the manuscript. Add a couple of sentences at an appropriate place.

We have added additional discussion of Fig 4A (now separated into panels A and B) as follows:

“When examining the gene content of these spots related to virulence, stress, antibiotic resistance, and defence systems, we can observe that genes with specific functions show a clear propensity to congregate within particular locations (**Fig. 4a**). For example, virulence genes tend to favour hotspots **##**21, **##**30, **##**92, and **##**94, while defence systems seem to prefer hotspots **##**22 and **##**68. However, the specific gene content of these spots can vary, as exemplified in **Fig. 4b** and detailed in **Fig. 4c**. Specific gene families also show preferences for particular spots. For instance, *lfp* genes preferentially localise in hotspot **##**30, while *fae* genes predominantly localise in spot #36 (**Fig. 4c, S12 Table**).”

Fig 4A contains 3 different figures, Change to 3 individual panels.

Figure 4A is split into two individual panels (A and B) in the revised manuscript.

Fig 4A : The first graph should be organised in a logical way, ie by ascending/descending numbers and spots vs hotspots. The second part shows a useful diagram of the S. Typhimurium 14028s genome with 5 spots/hotspots mapped. However the genome of strain LT2 was used as reference for mapping. The third part highlights certain spot/hotspot. I suggest expanding the third part as a sole panel or figure, with a diagram of the spots/hotspots that are most discussed in the manuscript (eg ##22, ##21, ##30, ##68, ##53, ##21, #63, #39, etc), including gene context. Unclear what the serovar names indicate.

In the revised manuscript, the spots in Fig. 4a have been organised in ascending order and aligned according to the spots and hotspots. *S*. Typhimurium 14028s was used as a reference for mapping instead of *S*. Typhimurium LT2. We appreciate the reviewer’s suggestion to expand the third part of Fig. 4a into a separate panel or figure, highlighting the hotspots most discussed in the manuscript. However, as we provide an interactive visualisation of the gene content of the top 26 spots on the Github associated with this manuscript (https://simrankushwaha.github.io/Genome-Plasticity-in-Salmonella), we decided not to create additional static figures. We have indicated the existence of this interactive visualisation in the figure legend for clarity. Additionally, we have added an explanation in the legend about the serovar names, which, along with the accession number, indicate the origin of the RGP.

**Fig 4. Distribution of gene content in the spots of *Salmonella* genomes.** **(A)** Frequency of pathogenicity determinants across spots identified in at least 1% strains of *Salmonella.* The number of strains containing each spot is indicated at the right of the bars. (**B**) Mapping of spots present on the reference strain *S. enterica* subsp. *enterica* serovar Typhimurium str. 14028s, with examples on the right illustrating various gene arrangements. The accession number, serovar and genomic location are provided. In panels (A) and (B), virulence factors, stress resistance genes, antibiotic resistance genes, defence systems, other RGP genes, and spot border genes are colour-coded according to the key. The category of other RGP genes represents those genes within the RGP that are not the specific pathogenic determinants under study. **(C)** Heatmap of the frequency at which a specific gene cluster appears outside a spot, in one of the 26 most abundant spots, or in other spots. Only gene clusters found in the 26 most abundant spots are depicted. The total count of gene cluster instances found across the *Salmonella* genus is shown on top. An interactive visualisation of the gene content in the spots can be found on the Github linked to this manuscript.

Fig 4A: The colour of the "spot border" is too similar to the colour of "virulence factor".

The spot border genes are now coloured in darker grey in the revised manuscript, to better distinguish from virulence factor genes.

Fig 4B: The "Gene present in spot (%)" colour scheme is the same as  "spot border" in Fig 4A. Change one of the colours to remove this confusion.

We have modified the colour scheme to avoid confusion.

Fig 4B lacks clarity due to a brief and vague legend. The legend states the "frequency at which a specific gene cluster appears outside a spot" but from the text suggests that those elements lie within said spots/hotspots. The Spots/hotspots should be organised in ascending/descending order and spots vs hotspots.

In the revised figure, the spots have been organised in ascending order and aligned according to the spots and hotspots. Furthermore, we improve the clarity of the legend (see legend in response to comment above) and added a section in the text discussing the elements that locate most frequently outside of a spot:

“In our study, we also observed that the majority of gene clusters are not associated with specific genomic spots (Fig. 4c). This lack of association can be attributed to several factors. Firstly, many gene clusters, including some responsible for stress resistance (e.g. qac, mer, ter) and antibiotic resistance (e.g. β-lactam, chloramphenicol, streptomycin, among others), are predominantly located on plasmids rather than chromosomal spots (S1 Fig). Plasmids often serve as vectors for horizontal gene transfer, contributing to their widespread distribution across different strains without a fixed genomic position. Additionally, virulence genes such as spv, fyu, irp, and ybt, along with defence systems like PifA, Gao_19, and Bunzi also frequently reside on plasmids, further explaining their absence from chromosomal spots (S1 Fig). Conversely, certain gene clusters, such as sop and ssp from SPI-1 (18) and defence systems RMII and RMIII (S1 Fig) are ubiquitous in Salmonella genomes. Their consistent presence across various strains likely precludes their identification in variable spots in our analysis.”

Fig 4B : What  do the numbers at the top indicate? The organisation of gene names, antimicrobials, and defence systems should follow a logical order. Add the gene names used to define the ABR categories in the legend.

The N at the top represents the count of the gene/system in the 12.2K *Salmonella* strains. We have added this information to the figure legend. The organisation of ABR and defence systems follows alphabetical order. The organisation of virulence factors and stress resistance follows alphabetical order within categories, as shown in Fig. S1. We have now displayed these categories also in Fig. 4. The gene names used to define the ABR categories are multiple, which is why they have not been depicted. However, these are shown on Table S10.

**“Fig 4. Distribution of gene content in the spots of *Salmonella* genomes.** **(A)** Frequency of pathogenicity determinants across spots identified in at least 1% strains of *Salmonella.* The number of strains containing each spot is indicated at the right of the bars. (**B**) Mapping of spots present on the reference strain *S. enterica* subsp. *enterica* serovar Typhimurium str. 14028s, with examples on the right illustrating various gene arrangements. The accession number, serovar and genomic location are provided. In panels (A) and (B), virulence factors, stress resistance genes, antibiotic resistance genes, defence systems, other RGP genes, and spot border genes are colour-coded according to the key. The category of other RGP genes represents those genes within the RGP that are not the specific pathogenic determinants under study. **(C)** Heatmap of the frequency at which a specific gene cluster appears outside a spot, in one of the 26 most abundant spots, or in other spots. Only gene clusters found in the 26 most abundant spots are depicted. The total count of gene cluster instances found across the *Salmonella* genus is shown on top. An interactive visualisation of the gene content in the spots can be found on the Github linked to this manuscript.”

S1 Fig: In the legend, what does "across Salmonella" mean? Change to "across the Salmonella genus".,

The change has been incorporated in the revised files.

S2 Fig: It is not clear what is being shown in this figure. Suggest that the figure legend is rewritten to explain that the figure is showing which virulence/antibiotic/stress/defence genes are carried on particular plasmids or prophages. The authors need to provide a supplementary table to allow readers to delve into the information in more detail.

We have modified the figure legend as suggested. The details can be found in the Supplementary Table S2, S4 and S9; this is now indicated in the legend.

**“S2 Fig. Distribution of pathogenicity determinants on prophage and plasmid classes.** **(A)** Prevalence of virulence factors, antibiotic resistance (ABR) genes, stress resistance genes, and defence systems on different prophage genera. **(B)** Prevalence of virulence factors, ABR genes, stress resistance genes, and defence systems on different plasmid incompatibility groups. For (A) and (B), prevalence values shown represent the relative percentages of pathogenicity factors found in prophages/plasmids, indicating the proportion of these factors present within each specific prophage/plasmid class. Detailed values are shown in Tables S2, S4, and S9.”
